# Molecular Research for Cereal Grain Quality

**DOI:** 10.3390/ijms241813687

**Published:** 2023-09-05

**Authors:** Jinsong Bao, Jian-Hong Xu

**Affiliations:** Hainan Institute, Zhejiang University, Yazhou Bay Science and Technology City, Yazhou District, Sanya 572025, China; jhxu@zju.edu.cn

Cereals such as wheat (*Triticum aestivum* L.), rice (*Oryza sativa* L.), and maize (*Zea mays* L.) provide key sources of dietary energy for human beings. Deficiency in their production will bring serious food security problems. Due to technical advances, for example, the utilization of heterosis in the development of hybrid cereals, many countries have achieved self-sufficiency in cereal production. However, due to increases in the human population and urbanization and a decline in arable land, the higher production demand remains challenging for many nations. On the other hand, with the increase in living standards thanks to economic development, our desire for a better life requires more production of high-quality cereal foods [1,2].

Cereal grain quality is governed by all the features and characteristics of the grain and its products to meet the demands of end users, which includes milling efficiency, processing quality, grain shape and appearance, ease of cooking, palatability, and nutrition [3]; it mainly reflects the physical and chemical properties of the grain. Physically, the grain’s shape and size affect the quality of appearance and also the yield, while chalkiness (especially for rice) affects its appearance and processing quality. Chemically, the major constituents of cereals, i.e., starch, protein, and lipids, affect cooking and eating quality, while protein, lipids, and other micronutrients affect nutritional quality [3]. All the physical and chemical properties are considered to be complex traits that are affected by both genetic and environmental factors [3]. However, molecular mechanisms underlying grain quality formation are poorly understood, which may constrain our ability to produce high-quality cereal grain. This Special Issue aims to provide a forum on the most recent advances in the application of molecular tools to understand the mechanism for improving any cereals’ grain quality. A total of 13 papers were collected for this Special Issue [1,2,4,5,6,7,8,9,10,11,12,13,14], mainly covering rice and wheat crops.

Rice is one of the most important staple food crops in the world and feeds more than half of the world’s population. The grain quality of rice generally includes the milling, appearance, cooking and eating, and nutritional qualities [3]. Liu et al. [4] cloned a novel quantitative trait locus (QTL), *GLW7.1* (*Grain Length*, *Width and Weight 7.1*), which encodes the CCT motif family protein GHD7. It was hypothesized that GHD7 participates in GA biosynthesis to increase grain size and is regulated by the GID1-GA-DELLA module as the feedback of the pathway. The near-isogenic line constructed with the dominant allele showed reduced chalkiness, improved cooking and eating quality, and increased grain length [4].

Rice cooking and eating quality is especially important as it directly affects consumer taste preferences and market values. The eating quality can be indirectly predicted with a series of starch physicochemical property evaluations [15]. Starch, including amylose and amylopectin, is synthesized with the actions of a series of enzymes, such as ADP-glucose pyrophosphorylase (AGPase), granule-bound starch synthase (GBSS), soluble starch synthases (SSs), starch branching enzymes (BEs), starch debranching enzymes (DBEs), and phosphorylases [5]. Amylose is synthesized by GBSS I, encoded by the *Waxy* gene (*Wx*). Apparent amylose content (AAC) is an important indicator to evaluate the eating quality of rice grains [15]. Starch gelatinization temperature (GT) is another important predictor to determine the cooking quality of rice. GT is mainly controlled by the *SSIIa/ALK* (*alkaline degenerate*) gene [1,15]. Fragrance is appealing to consumers, which is controlled by the recessive gene *fgr*/*OsBADH2 *(*betaine aldehyde dehydrogenase 2*) [6,7,15]. Pan et al. [1] analyzed the allelic diversification of the *Wx* and *ALK* loci in indica restorer lines developed over the last 50 years. The proportion of the *Wx^a^* allele decreased, while that of the *Wx^b^* allele increased, leading to a decrease in AAC and an increase in cooking and eating quality in current rice cultivars. *ALK* had no significant effect on taste value, and there was no strict requirement for the GT of high-quality rice, resulting in no selection pressure for *ALK* [1]. CRISPR/Cas9 technology was used to edit the 5′ UTR of the *Wx* gene of three lines with undesirably high AAC to create a batch of soft rice (low-AAC rice) breeding materials. The edited rice lines had an AAC of 16–18% and gel consistency of 77–80 mm, suggesting that the eating quality was successfully improved [2]. Lu et al. [6] obtained a high-quality chromosome-level genome assembly (~378.78 Mb) for a new fragrant japonica cultivar “Changxianggeng 1813”, with 31,671 predicted protein-coding genes. They found that it was the *badh2-E2* type of deletion (a 7 bp deletion in the second exon) that causes fragrance in this rice. The identification of many single-nucleotide polymorphisms, insertion-deletion polymorphisms (InDels), and large structure variants (SVs, >1000 bp) will be useful for genomics-assisted breeding in fragrant japonica rice. Tian et al. [7] edited *Wx* and *OsBADH2* simultaneously using CRISPR/Cas9 system to produce both homozygous two-line male sterile mutant lines and homozygous restorer mutant lines with free Cas9. The obtained mutants had a much lower AAC while having a significantly higher 2-acetyl-1-pyrroline aroma content. Based on this, a fragrant glutinous hybrid rice was developed without much effect on most agronomic traits.

Heading date may affect starch structure and grain yield due to different temperatures during seed development. Crofts et al. [8] constructed near-isogenic rice lines with *ss2a Hd1* (*heading date1*), *ss2a Hd1 hd1*, and *ss2a hd1* genotypes. They found that the *ss2a Hd1* line showed the highest plant biomass but with varied grain yield across different years. The *ss2a Hd1 hd1* line showed a higher total grain weight than *ss2a hd1*. The *ss2a hd1* line produced the lowest number of premature seeds and showed higher GT and lower AAC than *ss2a Hd1*, suggesting that *Hd1* is a candidate gene for developing high-yielding rice cultivars with the desired starch structure. Seed development and grain quality may also be affected by epigenetic regulation; DNA methylation is one of the main epigenetic modifications. Irshad et al. [9] generated a null mutant of a rice DNA demethylation gene, *Repressor Of Silencing 1a* (*OsROS1a*), with an in-frame deletion of the complete loss function of the Per-CXXC domain using CRISPR/Cas9 technology. The *osros1a* mutant showed longer and narrower grains, and seeds were deformed and contained an underdeveloped and less-starch-producing endosperm with slightly irregularly shaped embryos. The grains of the mutant were slightly opaque with rounded starch granules. RNA-Seq results indicated that the key genes for starch synthesis (*OsSSIIa* and *OsSSIIIa*) and cellulose synthesis (*CESA2*, *CESA3*, *CESA6*, and *CESA8*) and genes encoding polysaccharides and glutelin were downregulated in the mutant endosperm. Furthermore, 378 differential alternative splicing (AS) genes were identified in the mutant, suggesting that *OsROS1a* has an impact on AS events. Further analyzing the generated mutants, they produced a frameshift mutation to truncate the Pem-CXXC and RRMF domains of *OsROS1a*; this mutant had shrink spikelets, smaller anthers, and pollen grains and was not stained by iodine staining, showing a significant reduction in total soluble sugar and starch contents as compared to the wild type, which caused complete male sterility [10].

The enzymes to synthesize starch usually form a multienzyme complex, but whether these complexes change during seed development is not fully understood. Ying et al. [5] revealed that most of the enzymes except for SSIVb were eluted from GPC, first in smaller-molecular-weight fractions at the early developing stage, and then transferred to higher-molecular-weight fractions at the later stage in both WT and a *BEIIb* mutant (*be2b*). However, the inverse elution pattern of SSIVb may be attributed to its vital role in the initiation step of starch synthesis. The number of protein complexes was markedly decreased in *be2b* at all development stages as compared to those in BEIIb. Although SSIVb could partially compensate for the role of BEIIb in protein complex formation, it was difficult to form a larger protein complex containing over five proteins in *be2b*. These findings unraveled a dynamic change in the protein complex during seed development, which enables a deeper understanding of the complex mechanism of starch biosynthesis and quality improvement in rice.

Storage proteins represent the second-largest storage substance in cereal grains and play an important role in determining the nutritional as well as cooking and eating quality of cereals. In cereal endosperm cells, seed storage proteins are synthesized on the endoplasmic reticulum (ER), where they proteins are translocated to the lumen. The localization of mRNAs plays an essential role in governing gene expression and protein targeting and thus determines cell fate, development, and polar growth. Zhang et al. [11] reviewed the current knowledge of the mechanisms and functions of mRNA localization to the ER in cereal endosperm cells. mRNA targeting to ER subdomains is driven by specific RNA zipcodes and requires a set of trans-acting RBPs that recognize and bind these zipcodes and recruit other factors to mediate active transport. A more detailed network of cotransported mRNAs and the mechanism of assembly and remodeling of multi-RNA-binding protein (RBP) complexes to recognize and bind target mRNAs deserve further investigation.

The storage of rice is also an important part of its production and transactions, and only with good storage performance can its commercial value be maintained in commodity transactions. Zhu et al. [12] found that under high-temperature storage conditions (35 °C), the indica–japonica hybrid rice Yongyou 1540 was not significantly worse in terms of fatty acid value, whiteness value, and changes in electron microscope profile. Metabolomics analysis identified 19 key differential metabolites, in which the lipid metabolites related to palmitoleic acid were found to affect the aging of rice. In addition, two substances, guanosine 3′,5′-cyclophosphate and pipecolic acid, were beneficial to enhancing the resistance of rice to harsh storage conditions, thereby delaying the deterioration of, and maintaining, its quality.

Wheat grain quality mainly includes processing and nutritional quality. The processing quality mainly depends on the content and characteristics of storage proteins, which are important indicators for market value and consumer acceptance. Cao et al. [13] identified two novel high-molecular-weight glutenin subunits (HMW-GS) 1Ax2.1* at *Glu-A1* and 1By19* at *Glu-B1* from German spelt wheat with 2478 bp and 2163 bp encoding 824 and 720 amino acid residues, respectively. The specific single-nucleotide-polymorphism-based markers for 1Ax2.1* and 1By19* genes were developed and validated by using a wide range of wheat accessions, which provide new gene resources and molecular markers for improving wheat’s breadmaking quality.

Spring cold stress (SCS) causes a serious threat to wheat reproductive tissues and grain production, which is a major constraint in achieving high grain yield and quality in winter wheat. Su et al. [14] reviewed the physiological and molecular mechanisms involved in wheat floret and spikelet SCS tolerance and summarized QTLs that regulate SCS to identify candidate genes for breeding. To sustain grain setting and quality under SCS, it is necessary to breed novel wheat cultivars using novel SCS-tolerant QTLs or genes with regards to floret and spikelet development in new breeding strategies and uncover the fundamental resistance mechanism.

All these papers provide new scientific insights into molecular research for cereal grain quality. However, most of them focus on the grain quality of rice and wheat crops, so information on other important cereals, including maize, oat, barley, millets, sorghum, rye, etc., is still limited. We hope to collect papers with a broad coverage of the grain quality features of more cereal crops in future Special Issues.
